# Supplementary material for: Seminoma and Embryonal Carcinoma Footprints Identified by Analysis of Integrated Genome-Wide Epigenetic and Expression Profiles of Germ Cell Cancer Cell Lines
Source: PLoS One. 2014 Jun 2;9(6):e98330. doi: 10.1371/journal.pone.0098330 (PMC4041891; doi:10.1371/journal.pone.0098330)
Supplement: File S1 — ZIP file containing DMRforPairs output for significant regions. Please start from the html files. (ZIP) [file pone.0098330.s008.zip › figures/33772.pdf]

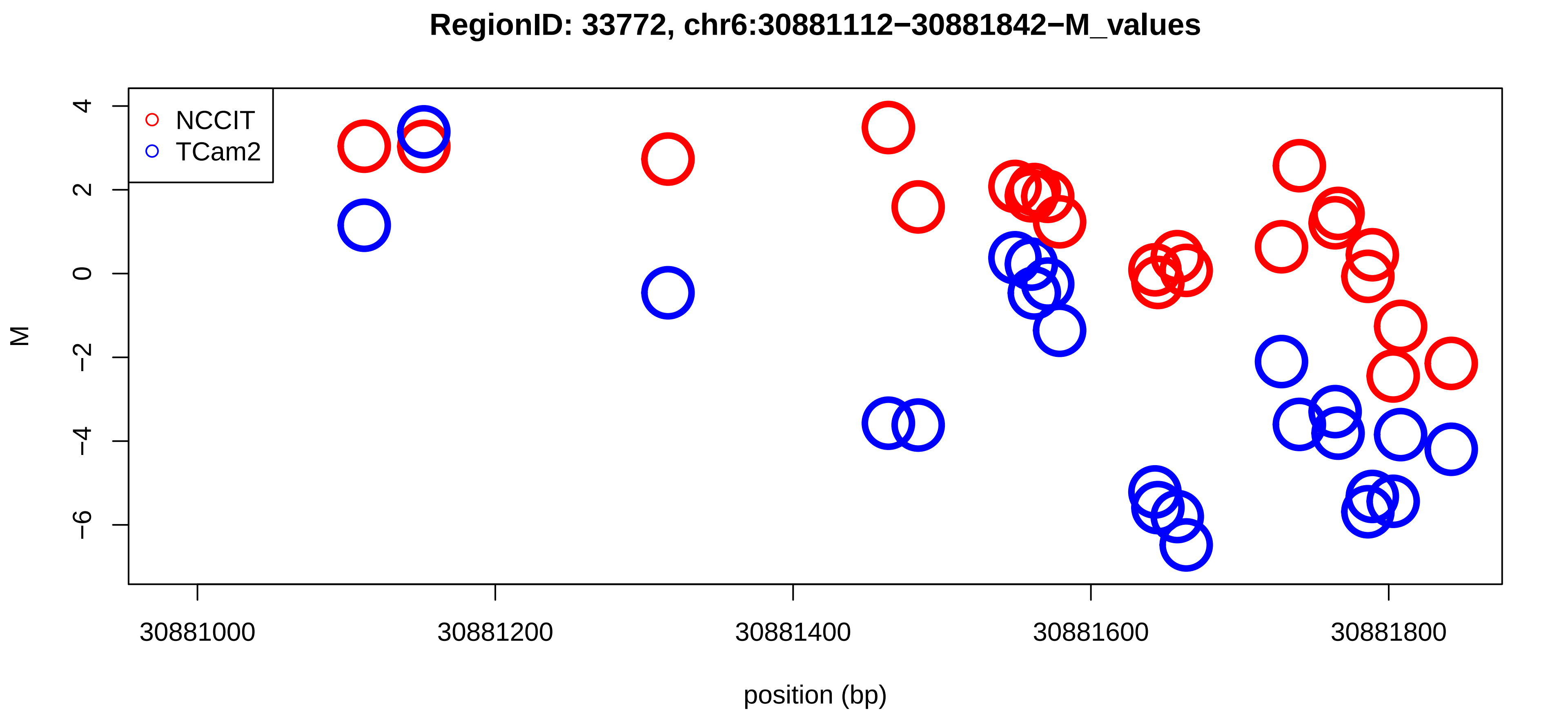
RegionID: 33772, chr6:30881112−30881842−M_values
NCCIT
TCam2
M
4
2
0
−2
−4
−6
30881000
30881200
30881400
30881600
30881800
position (bp)

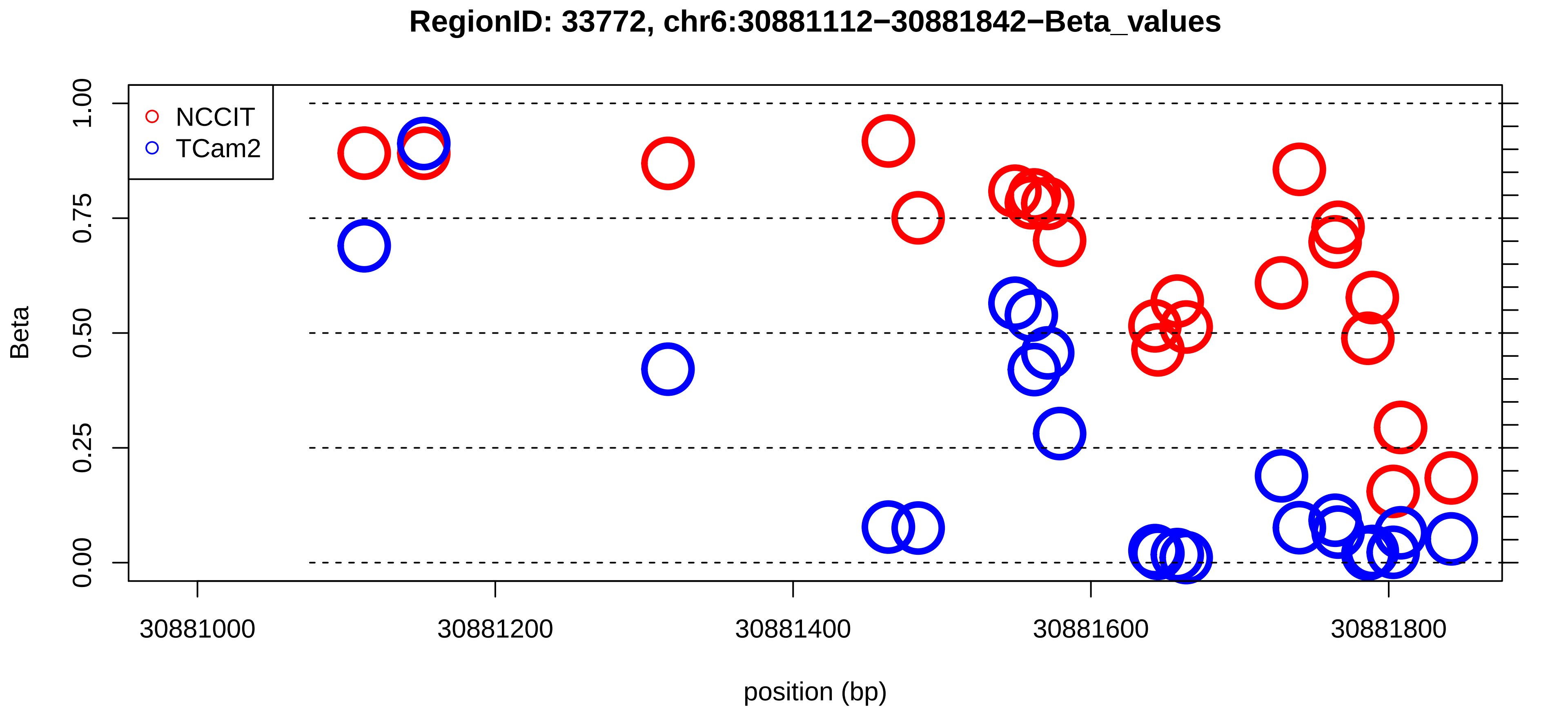
RegionID: 33772, chr6:30881112−30881842−Beta_values
NCCIT
TCam2
Beta
1.00
0.75
0.50
0.25
0.00
30881000
30881200
30881400
30881600
30881800
position (bp)

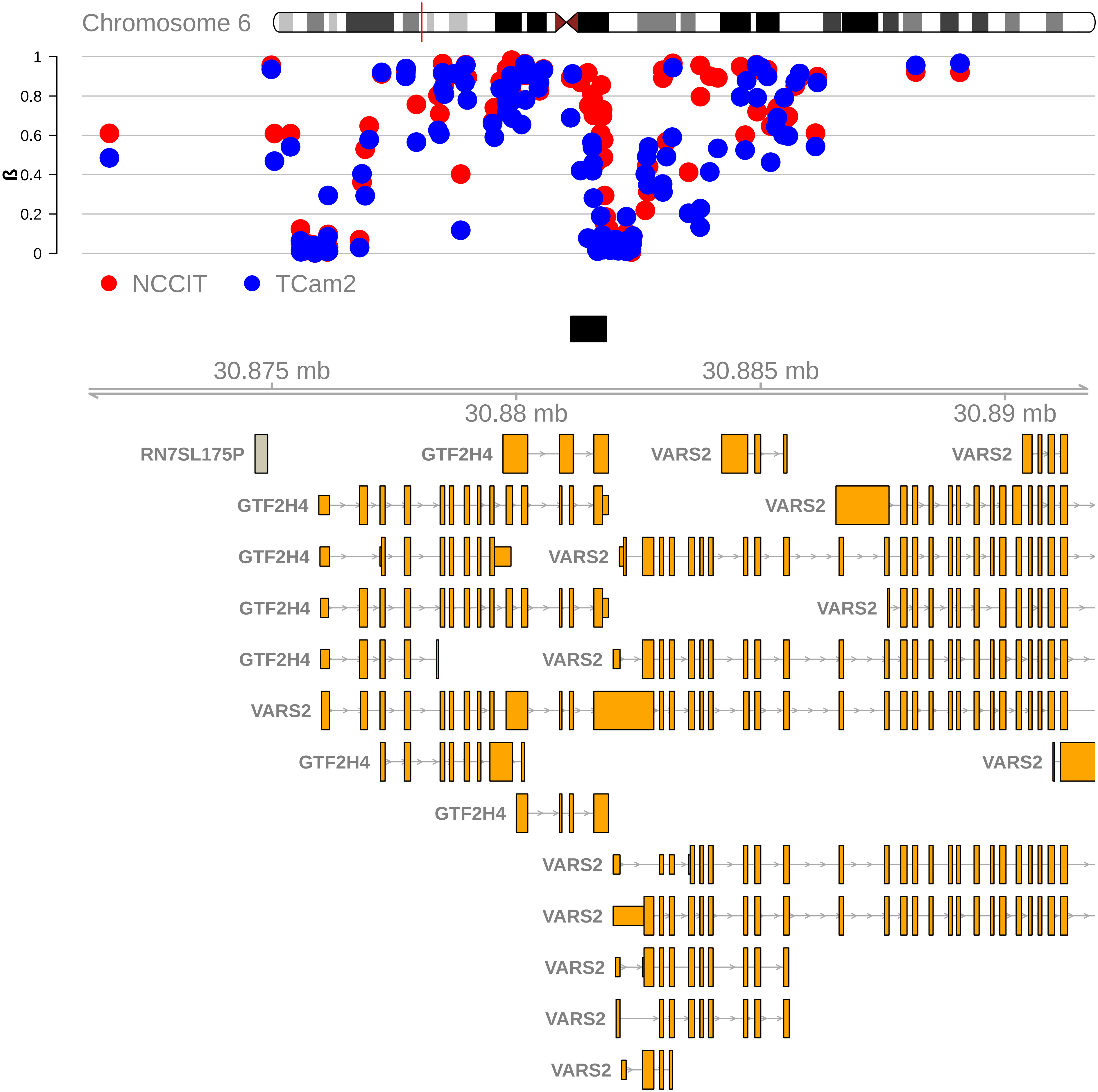

Chromosome 6
1
0.8
0.6
ß 0.4
0.2
0
NCCIT
TCam2
30.875 mb
30.885 mb
30.88 mb
30.89 mb
RN7SL175P
GTF2H4
VARS2
